# Development of weight and age-based dosing of daily primaquine for radical cure of vivax malaria

**DOI:** 10.1186/s12936-021-03886-w

**Published:** 2021-09-09

**Authors:** Walter Robert Taylor, Richard M. Hoglund, Pimnara Peerawaranun, Thuy Nhien Nguyen, Tran Tinh Hien, Arnaud Tarantola, Lorenz von Seidlein, Rupam Tripura, Thomas J. Peto, Arjen M. Dondorp, Jordi Landier, Francois H.Nosten, Frank Smithuis, Koukeo Phommasone, Mayfong Mayxay, Soy Ty Kheang, Chy Say, Kak Neeraj, Leang Rithea, Lek Dysoley, Sim Kheng, Sinoun Muth, Arantxa Roca-Feltrer, Mark Debackere, Rick M. Fairhurst, Ngak Song, Philippe Buchy, Didier Menard, Nicholas J. White, Joel Tarning, Mavuto Mukaka

**Affiliations:** 1 Mahidol Oxford Tropical Medicine Research Unit, Faculty of Tropical Medicine, Mahidol University, 420/60 Rajvithi Road, Bangkok, 10400 Thailand; 2 Centre for Tropical Medicine and Global Health, Nuffield Department of Medicine, University of Oxford, Oxford, UK; 3 Oxford University Clinical Research Unit, Wellcome Trust Major Oversea Programme, Ho Chi Minh City, Vietnam; 4 Institut Pasteur du Cambodge, 5 Monivong Boulevard, Phnom Penh, 12201 Cambodia; 5 Department of Global Health, Amsterdam University Medical Center, Amsterdam, The Netherlands; 6 Shoklo Malaria Research Unit, Mae Sot, Thailand; 7 Aix-Marseille Université, IRD, INSERM, SESSTIM, Marseille, France; 8 Myanmar Oxford Clinical Research Unit, Yangon, Myanmar; 9 Lao-Oxford-Mahosot Hospital-Wellcome Trust Research Unit, Mahosot Hospital, Vientiane, Lao PDR; 10 Amsterdam Institute for Global Health & Development, Amsterdam, The Netherlands; 11 Institute of Research and Education Development, University of Health Sciences, Vientiane, Lao PDR; 12 Center for Health and Social Development (HSD), National Institute for Public Health (NIPH) and University Research Co., LLC (URC), Chey Chumneas, Daun Penh, Phnom Penh, Cambodia; 13 AQUITY Global Inc, 987 Avenel Farm Dr, Potomac, MD 20854 USA; 14 University Research Co., LLC Washington DC, 7200 Wisconsin Ave, Bethesda, MD 20814 USA; 15 National Center for Parasitology, Entomology and Malaria Control, Khan Sen Sok, Phnom Penh, Cambodia; 16 Institute of Public Health, Phnom Penh, Cambodia; 17 Malaria Consortium, London, UK; 18 MSF Belgium Cambodia Malaria Program, Khan Chamkarmon, Phnom Penh, Cambodia; 19 Laboratory of Malaria and Vector Research, National Institute of Allergy and Infectious Diseases, National Institutes of Health, Rockville, MD 20852 USA; 20 FHI 360 Cambodia Office, Keng Kang III Khan Chamkamon, Phnom Penh, Cambodia; 21 GSK Vaccines, 23 Rochester Park, Singapore, Singapore; 22 Unité Génétique du Paludisme Et Résistance, Département Parasites Et Insectes Vecteurs, Institut Pasteur, Paris, France

**Keywords:** Primaquine, Allometric scaling, Age-based dosing, Weight-based dosing, *Plasmodium vivax*

## Abstract

**Background:**

In many endemic areas, *Plasmodium vivax* malaria is predominantly a disease of young adults and children. International recommendations for radical cure recommend fixed target doses of 0.25 or 0.5 mg/kg/day of primaquine for 14 days in glucose-6-phosphate dehydrogenase normal patients of all ages. However, for many anti-malarial drugs, including primaquine, there is evidence that children have lower exposures than adults for the same weight-adjusted dose. The aim of the study was to develop 14-day weight-based and age-based primaquine regimens against high-frequency relapsing tropical *P. vivax*.

**Methods:**

The recommended adult target dose of 0.5 mg/kg/day (30 mg in a 60 kg patient) is highly efficacious against tropical *P. vivax* and was assumed to produce optimal drug exposure. Primaquine doses were calculated using allometric scaling to derive a weight-based primaquine regimen over a weight range from 5 to 100 kg. Growth curves were constructed from an anthropometric database of 53,467 individuals from the Greater Mekong Subregion (GMS) to define weight-for-age relationships. The median age associated with each weight was used to derive an age-based dosing regimen from the weight-based regimen.

**Results:**

The proposed weight-based regimen has 5 dosing bands: (i) 5–7 kg, 5 mg, resulting in 0.71–1.0 mg/kg/day; (ii) 8–16 kg, 7.5 mg, 0.47–0.94 mg/kg/day; (iii) 17–40 kg, 15 mg, 0.38–0.88 mg/kg/day; (iv) 41–80 kg, 30 mg, 0.37–0.73 mg/kg/day; and (v) 81–100 kg, 45 mg, 0.45–0.56 mg/kg/day. The corresponding age-based regimen had 4 dosing bands: 6–11 months, 5 mg, 0.43–1.0 mg/kg/day; (ii) 1–5 years, 7.5 mg, 0.35–1.25 mg/kg/day; (iii) 6–14 years, 15 mg, 0.30–1.36 mg/kg/day; and (iv) ≥ 15 years, 30 mg, 0.35–1.07 mg/kg/day.

**Conclusion:**

The proposed weight-based regimen showed less variability around the primaquine dose within each dosing band compared to the age-based regimen and is preferred. Increased dose accuracy could be achieved by additional dosing bands for both regimens. The age-based regimen might not be applicable to regions outside the GMS, which must be based on local anthropometric data. Pharmacokinetic data in small children are needed urgently to inform the proposed regimens.

**Supplementary Information:**

The online version contains supplementary material available at 10.1186/s12936-021-03886-w.

## Background

Primaquine and tafenoquine are the only registered drugs recommended currently to kill *Plasmodium vivax* and *Plasmodium ovale* hypnozoites (dormant parasites residing in the liver) and prevent relapses, which are fresh blood-stage infections that arise periodically from hypnozoites. Although primaquine has been available since the 1950s, there are no internationally recommended practical regimens that detail the dose to be given for a given weight band in patients with normal (G6PDn) or deficient (G6PDd) glucose-6-phosphate dehydrogenase activity.

The World Health Organization (WHO) recommends 0.5 mg/kg/day for 14 days (total dose of 7.0 mg/kg) for South East Asia and Oceania and 0.25 mg/kg/day for 14 days (total dose of 3.5 mg/kg) elsewhere [1, 2]. Weekly primaquine at 0.75 mg/kg for 8 weeks is recommended for G6PDd patients in all regions. In the absence of established dosing regimens, researchers and malaria control programmes (MCPs) have used many different weight- and age-based regimens, adapting the recommended weight-based target doses to their populations [3] (Additional file 1: Table S1). Complex regimens with multiple dosing bands (20 and 21 in two trials 12 [4, 5]) result in more accurate dosing but are too cumbersome to administer outside of the research setting. Different primaquine regimens might result in under- or over dosing in particular patient groups so an evidence-based approach is essential to develop primaquine regimens.

Moreover, getting the dose right is crucial because the antirelapse efficacy of primaquine depends on the total exposure to primaquine [6, 7]. However, fixed weight-based target doses across ages often results in underexposure in small children due to their greater weight-normalised clearance (L/h/kg) compared to older children and adults. This results in a nonlinear relationship between drug exposure and body weight at a specific weight-based target dose [8]. Limited data on single low dose primaquine (SLDPQ), dosed at 0.25 or 0.4 mg/kg, administered on Day 2 in artemether–lumefantrine treated *Plasmodium falciparum*-infected African children aged 2–14 years, showed that primaquine and carboxyprimaquine exposures were lower in young children (i.e. low body weight) [9]. Based on their pharmacokinetic model, Goncalves et al*.* predicted approximately half the peak primaquine concentrations in a 2-year old child (12 kg) compared to a 14-year old child (40 kg) after receiving 0.25 mg/kg of primaquine (~ 30 ng/mL vs. ~ 73 ng/mL and showed increasing exposure to primaquine and carboxyprimaquine with increasing weight and age. The lack of pharmacokinetic data in young children with vivax malaria is a major evidence gap. If the trends to lower exposures at younger age observed in African children given SLDPQ are confirmed then dose regimens based largely on adult data will need to be revised.

Dosing by age is intrinsically less accurate than dosing by body weight because of the high degree of variability of bodyweight for a given age, but it has the advantage of not requiring weighing scales, which is desirable in mass drug administrations, the informal health system and remote areas [10–12]. The aim of primaquine therapy is to achieve high efficacy and good tolerability within acceptable therapeutic ranges, irrespective of the dosing strategy (age vs*.* bodyweight dosing).

There is good evidence, mostly in adults, of the high efficacy of radical cure primaquine against tropical *P*. *vivax* with a total target dose of 7 mg/kg [4, 5, 13, 14] and a fall in efficacy with lower doses [15–17]. Modest antirelapse efficacy of 58%, relative to no primaquine, was reported for the 7 mg/kg regimen in children aged 1–5 years from Papua New Guinea [18], a high risk group for relapse [19]. There are insufficient data to define a minimally effective total dose in children < 5 years but in adults and likely in children aged ≥ 5 years [20], this is probably ~ 5.25 mg/kg. This dose resulted in relapse rates of 0%, ~ 2% and ~ 7% in volunteers with experimentally-induced Chesson strain of *P. vivax* [17], in Thai patients [21], and in Australian soldiers [22], respectively. By contrast, the 3.5 mg/kg total dose, which is recommended by some GMS countries, was associated with variable relapse rates ranging from ~ 7 to 25% [21, 23–25] and was 50% in adults with Papuan-acquired *P. vivax* (K. Baird, pers. commun.).

Abdominal pain, methaemoglobinaemia and acute haemolysis in G6PD deficient individuals are dose-dependent primaquine toxicities but G6PD status per se does not affect primaquine pharmacokinetics [26]. Abdominal pain is reduced by food [27, 28], which also increases primaquine exposure [29]. Rates of abdominal pain in the IMPROV study (median age of 16 years) were significantly higher in the 1.0 vs. 0.5 mg/kg/day arm (43% vs. 34%, p < 0.0001) and placebo (29%, p < 0.0001), while rates of early vomiting were remarkably low (~ 2% in all three arms) and only 5/935 (0.53%) of 1.0 mg/kg recipients stopped primaquine temporarily because of gastrointestinal toxicity [4]. Similarly, Chu et al*.* reported rates of abdominal pain of 33% vs*.* 27% and 20% vs*.* 10% (1.0 vs. 0.5 mg/kg/day) in chloroquine and dihydroartemisinin-piperaquine recipients (median age of 20 years), respectively. No patients stopped their primaquine early [5]. In both studies, children < 5 years represented only 4% [5] and 6% [4] of patients.

Methaemoglobinaemia varies considerably between individuals and is well tolerated in healthy individuals and malaria patients even when receiving the 1.0 mg/kg dose [5, 30, 31]. The median methaemoglobinaemia was 7.5% in the 1.0 mg/kg/day arm with 4/327 (1.2%) stopping their primaquine early [5]. Clinically significant, primaquine-induced haemolysis is greater in G6PD deficient hemizygous males and homozygous females compared to G6PD heterozygous females [32]. In Thailand, G6PDd heterozygous females receiving 1.0 mg/kg/day of primaquine had a greater mean fractional fall in haematocrit vs. those receiving 0.5 mg/kg/day (23.6 vs. 15.5%, p = 0.001) and two females in the former group were transfused [33]. Taken together, these data demonstrate the better tolerability of the 0.5 mg/day dose, but are insufficient to define with confidence a maximally tolerated dose.

Following a request from a malaria control programme for a practical dosing regimen of primaquine for radical cure, it was decided to develop a weight-based regimen and explore the possibility of an age-based regimen.

## Methods

### Anthropometric data

An anthropometric database was assembled, using data provided by researchers (malaria patients, clinic data), and the Demographic Health Survey (DHS), collected from large household, nationally representative survey data of mostly healthy individuals, from GMS countries (n = 54,660). As done previously [34, 35], a waiver for ethical review was granted by the Oxford Tropical Ethics Committee for analysing these anonymized data.

**Fig. 1:**
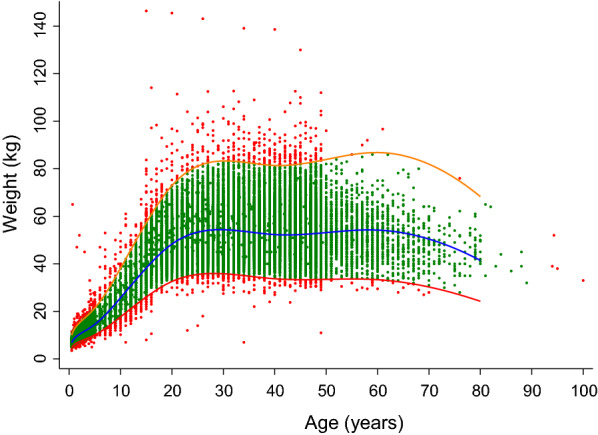
Distribution of the individual anthropometric weight-for-age data in the Greater Mekong Subregion for both sexes combined. The blue line represents the median when applying a three-parameter Box-Cox power exponential distribution and cubic spline smoothing, while the red and orange lines represent the 1st and 99th percentiles, respectively, of the model. The pink markers show outliers outside the 98% prediction interval of the model (n = 1193), and the green markers represent the observed data retained in the final model (n = 53,467)

The raw weight-for-age data were modelled into growth curves (Fig. 1 & Additional file 2: Figures S1 & S2) to define the relationship between weight and age [36] using a three-parameter Box-Cox power exponential distribution [37] and cubic spline smoothing [38]. Outliers were excluded from the database by removing data outside the 1^st^ and 99^th^ percentiles (n = 1193), resulting in a total number of 53,467 patients in the final database.

### Weight-based dosing regimen

Consistent with WHO recommendations and the strong evidence base for high efficacy of the 0.5 mg/kg daily dose in tropical *P. vivax*, the primaquine target dose was set to 0.5 mg/kg/day (i.e. 30 mg in a 60 kg patient, resulting in the target primaquine exposure). A theoretical dose for each bodyweight was calculated using allometric scaling (Eq. 1) with the aim of achieving comparable drug exposure in all patients.

1 $$\mathrm{Theoretical\,dose}\left(\mathrm{mg}\right)= 30\mathrm{ mg}\times \left(\frac{\mathrm{BW}}{60\mathrm{ kg}}\right)^{0.75}$$

BW is the individual body weight (kg) and 30 mg is the optimal dose in a 60 kg patient. Five primaquine doses were chosen (i.e. 5, 7.5, 15, 30, and 45 mg) to develop a programmatically feasible primaquine regimen with the current dosing strengths.

Each body weight was assigned to the dose that minimized the residuals between the calculated theoretical dose and the chosen dose. The fifth dosing band (i.e. 45 mg) was added specifically to address under-dosing in heavier patients (> 80 kg).

### Age-based dosing

The observed weight-for-age data in the final data base was used to calculate the median age for each body weight (1 kg increments). The age-based dosing was developed by replacing the weight bins in the weight-based regimen with the median age for each weight. Thus, it was not possible to separate heavier adults (> 80 kg) from standard-weight adults (41–80 kg) in the age-based dosing. This resulted in a total of four age-based dosing bands. The predicted mg/kg dose for each age was then calculated.

### Pharmacokinetic simulations

Simulations of primaquine and carboxyprimaquine exposures were carried out using a previously published pharmacokinetic model [39] and a simulation dataset. In the pharmacokinetic model, drug absorption was characterized by five-transit absorption compartments, with pre-systemic metabolism of primaquine to carboxyprimaquine. Primaquine and carboxyprimaquine were characterised by one-compartment disposition models. The model included allometrically-scaled body weight as a covariate with the exponents fixed to 0.75 for clearance parameters and 1 for volume parameters. These scaling exponents were used also in two previously published primaquine studies [9, 20]. Furthermore, the 0.75 exponent is used widely in paediatric dosing to compensate for the increased total drug clearance/unit weight in children, which commonly peaks at age 2 and is followed by a slow exponential decline [40, 41].

The model did not adjust for CYP2D6 metabolic status or use an enzymatic maturation factor to compensate for age-dependent drug clearance in very young children. CYP2D6-related poor metabolic activity is associated with poor primaquine efficacy [42, 43] and has a low prevalence rate (~ 1–9%) in South East Asia, contrasting with the high rates (40–60%) of the *10 genotype, which confers intermediate primaquine metabolism [44–46]. Given the high efficacy rates of the 0.5 mg/kg/day dose in tropical *P*. *vivax* in South East Asia, the effect of intermediate primaquine metabolic status appears small. A maturation factor for clearance is advocated by some authorities to take into account the immaturity of metabolizing enzymes and reduced renal function, seen mostly in neonates and infants [8, 47]. However, maturation of the clearance function was not included in the original published model [39] and more data would be needed to confirm the pharmacokinetic properties of primaquine in neonates and infants.

A simulation dataset was constructed from the observed anthropometric weight-for-age data (n = 54,660) by excluding pregnant women, patients above 50 years of age, and patients weighing < 5 kg and > 100 kg. The data in pregnant women were excluded as the model was not developed for this population and the relationship between age and weight might be different in this group. The limitation in weight and age was due to the limited amount of data outside the included intervals (5–100 kg and 0–50 years), which could make simulations in these groups of patients uncertain and unreliable. The data were then divided into 1 kg bins, and the data below the 1st and above the 99th percentile was removed from each bin, resulting in a simulation dataset of 50,872 individuals.

The developed weight-based and age-based dosing regimens were used to simulate a total of 1000 single dose exposures of primaquine in each of the 50,872 individual patients in the simulation dataset (i.e. a total of 50,872,000 simulated patients in each dose regimen) in NONMEM v7.3 (Icon Development Solutions, Ellicott City, Maryland, USA). Exposure up to 60 h post-dose was calculated based on these simulations, and visualised as the 2.5th, 50th and 97.5th percentiles, using R v4.0.0 (the R Foundation for Statistical Computing, Vienna, Austria).

## Results

### Database description

The modelled weight-for-age database contained 53,467 individuals with a median age and weight of 23 years (IQR 9—35) and 46.3 kg (IQR 21.5–54.1 kg). Females (36,733/53,467, 68.7%) outnumbered males and most individuals were healthy (86.0%, 45,949/53,467); 6244/53,467 (11.7%) had malaria (Table 1). Median weights were similar in males and females for all age groups, but with a greater difference in older individuals aged ≥ 15 years (males weighing a median of 3.4 kg more than females). Median weights were also similar between healthy individuals and those with malaria or other infections (Table 2 and Additional file 3: Figure S3).

**Table 1:** Summary of anthropometric weight-for-age data in the Greater Mekong Subregion

Characteristics, n (%)	Total^a^ (N = 53,467)	Male (n = 16,388)	Female (n = 36,733)
Country			
Cambodia	29,867 (55.9)	10,334 (63.1)	19,189 (52.2)
Myanmar	18,577 (34.7)	3117 (19.0)	15,460 (42.1)
Vietnam	3412 (6.4)	2107 (12.9)	1303 (3.6)
Laos	1611 (3.0)	830 (5.1)	781 (2.1)
Age groups (years), median (IQR) [range]	23 (9–35) [0.5–89]	14 (4–28) [0.5–83]	26 (16–37) [0.5–89]
< 1^b^	1063 (2.0)	537 (3.3)	526 (1.4)
1–5	10,296 (19.3)	5256 (32.1)	5027 (13.7)
6–14	4722 (8.8)	2503 (15.3)	2187 (6.0)
≥ 15	37,386 (69.9)	8092 (49.4)	28,993 (78.9)
Weight (kg), median (IQR) [range]	46.3 (21.5–54.1) [5.0–86.0]	36.0 (13.2–53.0) [5.1–86.0]	47.2 (39.0–54.5) [5.0–82.9]
> 70 kg	1320 (2.5)	229 (1.4)	1086 (3.0)
> 75 kg	499 (0.9)	69 (0.4)	428 (1.2)
Health status			
Healthy	45,949 (86.0)	10,988 (67.1)	34,941 (95.1)
Malaria	6244 (11.7)	4981 (30.4)	1245 (3.4)
Other infections	1247 (2.3)	393 (2.4)	546 (1.5)
No diagnosis	13 (< 0.1)	13 (0.1)	0 (0.0)

^a^Sex at birth undocumented in 346 individuals

^b^Age 6 to 11 months

**Table 2:** Weight as a function of sex and disease status

Age groups (years)	Total N	Weight (kg), median (IQR)	
	Male–female	Male	Female
< 1^a^	1063 (537–526)	8 (7.3–8.6)	7.5 (6.9–8)
1–5	10,283 (5256–5027)	12 (10.2–13.9)	11.5 (9.9–13.2)
6–14	4690 (2503–2187)	23 (19–29)	23 (18–30)
≥ 15	37,085 (8092–28,993)	53.4 (49–59)	50 (45–56.6)

	Healthy-malaria^b^	Healthy	Malaria^b^
< 1	1063 (1037–26)	7.7 (7–8.4)	7.7 (7–8)
1–5	10,295 (9613–682)	11.9 (10–13.6)	11.5 (10–13)
6–14	4721 (3195–1526)	23 (18.1–29)	24 (19–30)
≥ 15	37,374 (32,104–5270)	50.5 (45.2–57)	53 (48–58)

^a^Age 6 to 11 months

^b^Malaria and miscellaneous infections

The relationship between weight and age was described by a three parameter model, denoted by Box-Cox power exponential distribution ($\theta , \delta , \lambda$) where $\theta$ is the median, $\delta$ is the scale parameter, and $\lambda$ is the skewness parameter. We obtained The following parameter estimates were obtained after fitting the model; $\widehat{\theta }$ = 40.3 (95% CI 40.2–40.5); $\widehat{\delta }$ = 0.1707 (95% CI 0.1706–0.1708); $\widehat{\lambda }=-0.183$. The $\lambda$ (skewness parameter) is a constant and, therefore, no corresponding confidence interval can be estimated.

### Allometrically scaled weight-based regimen

**Fig. 2:**
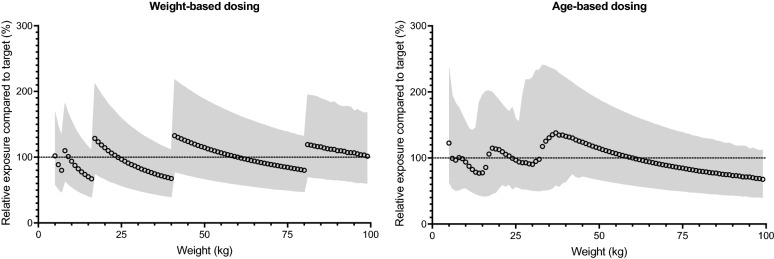
Simulated primaquine exposures, relative to the median exposure following administration of 30 mg primaquine base in a 60 kg patient (i.e. target exposure) for the developed weight-based and age-based dosing regimens. The shaded areas represent the 2.5th and 97.5th percentiles of the simulated exposures

**Fig. 3:**
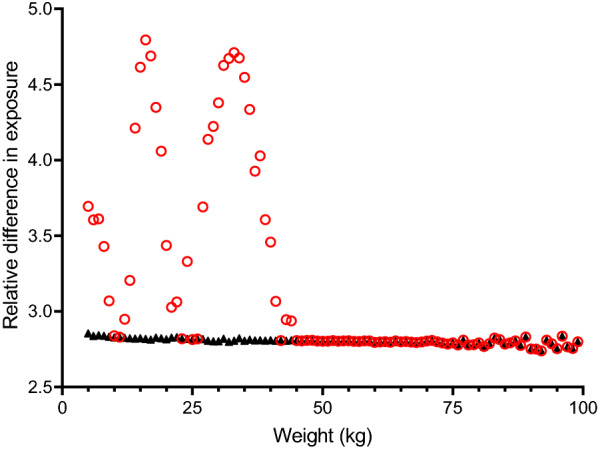
Relative variability in simulated exposure after different dosing regimens. Markers illustrate the relative difference between the simulated 2.5th and 97.5th percentiles of primaquine exposure, stratified on bodyweight. Variability associated with bodyweight-based dosing is shown as black triangles and variability associated with age-based dosing is shown as red circles

The proposed regimen has five dosing bands, covering weights from 5 to 100 kg (Table 2). Simulated primaquine exposures, after applying the proposed weight-based dosing, resulted in evenly distributed exposures around the target exposure (Fig. 2), with a maximum of a 2.85-fold difference between the lowest and highest exposure (i.e. 2.5–97.5 percentile of simulated exposures) within a body weight (Fig. 3). The predicted median primaquine exposure (AUC_0-60_) in a 60 kg individual was approximately 1200 h × ng/mL.

### Age-based dosing regimen

The proposed age-based regimen has four dosing bands starting at 6 months of age (Table 3); the resulting mg/kg dose for each age is shown in Additional file 4: Figure S4 and Additional file 5: Table S2. Patients aged 15 years were predicted to receive low doses if assigned 15 mg (median of 0.34 mg/kg) compared to 30 mg (median of 0.68 mg/kg); therefore, they were allocated 30 mg of primaquine. Simulated primaquine exposures, after applying the proposed age-based dosing, resulted in a substantially higher variability around the target exposure compared to the weight-based dosing (Figs. 2 and 3). A maximum of a 4.79-fold difference was seen between the lowest and highest exposure (i.e. 2.5–97.5 percentile of simulated exposures) within a bodyweight (Fig. 3).

**Table 3:** Proposed weight-based and age-based dosing of primaquine

Weight-based dosing			Age-based dosing		
Bodyweight (kg)	Primaquine daily dose^a^ (mg)	Primaquine daily dose^a^ (mg/kg/day)	Age (years)	Primaquine daily dose^a^ (mg)	Primaquine daily dose^a^ (mg/kg/day)
5–7	5	0.71–1.00	< 1^b^	5	0.43–1.00
8–16	7.5	0.47–0.94	1–5	7.5	0.34–1.25
17–40	15	0.38–0.88	6–14	15	0.28–1.30
41–80	30	0.37–0.73	≥ 15	30	0.35–1.11
81–100	45	0.45–0.56			

^a^Daily dose based on a 14-day regimen

^b^The lowest age-band is proposed for infants is 6 months to 11 months of age

## Discussion

This analysis has shown that a weight-based regimen can be designed within the parameters set by a MCP in the GMS, using available tablet strengths and designing dosing bands to avoid tablet fractions. Although the GMS population is generally slender, a fifth weight-based dosing band was essential to avoid under-dosing and low efficacy in heavier patients [7, 22, 48]. A 4-band, age-based regimen was successfully derived from the weight-based regimen but, as expected, resulted in greater variability in expected exposures and would be considered suitable only if weight-based dosing cannot be implemented.

MCPs, striving to eliminate *P. vivax* malaria, need tolerable, effective and user-friendly regimens. Only primaquine or tafenoquine are available to be deployed currently but both require G6PD testing before use and tafenoquine cannot be given to patients with < 70% G6PD enzyme activity. This will exclude a substantial number of patients, especially G6PD deficient heterozygous women [49]. Thus, there is still an urgent need to develop an optimized dosing regimen of primaquine for programme deployment.

Primaquine has several challenges, including adherence to 14-day [19, 50] and even 7-day regimens [51]. The limited availability of tablet strengths that are made according to international good manufacturing practice, 7.5 and 15 mg, leads to inaccurate, impractical regimens necessitating tablet fractions. Owing to the paucity of pharmacokinetic data, especially in malaria-infected children, who experience reduced drug exposure [3, 9], there are no WHO-approved, evidenced-based, allometrically-scaled, weight-based dosing regimens for radical cure.

Therefore, the aim of this study was to design such a dosing regimen using a detailed, published, structural pharmacokinetic model based on adult data [39]. The predicted total, primaquine exposure from this model was 1200 h × ng/mL after 30 mg of primaquine in a 60 kg patient (the predefined optimal dose), consistent with the value of 521 h × ng/mL obtained from 15 mg in Thai patients [26] and 610 h × ng/mL predicted from a pharmacokinetic model of healthy, non-obese Korean adults given 15 mg of primaquine with hydroxychloroquine [52]. Moreover, the allometrically-scaled, weight-based regimen predicted similar primaquine exposures across all weight groups with a < 3-fold difference in exposure.

Goncalves et al. reported that their allometrically-scaled pharmacokinetic model overestimated primaquine and carboxyprimaquine exposures in their falciparum-infected children, before implementing an age-related covariate on the relative bioavailability of primaquine (i.e. accounting for increased relative bioavailability with increasing age). Their model also showed increasing primaquine and carboxyprimaquine exposure with decreasing CYP2D6 metabolic activity.

The model used in the simulations reported here did not include an enzymic maturation factor, nutritional or CYP2D6 metabolizer status. The inclusion of an enzymatic maturation factor in the current pharmacokinetic model could lead to higher primaquine exposure in very young children (< 2 years) than predicted from the simulations. This should have no effect on the efficacy of primaquine in this group, but might need to be considered in terms of safety. Further studies are needed to fully characterize the effect of enzyme maturation on primaquine pharmacokinetics before this can be taken into consideration for this group of patients.

Another factor that could affect the dose regimen is severe acute malnutrition, which has been shown to reduce lumefantrine exposure [53]. How nutritional status affects primaquine pharmacokinetics and whether the proposed dosing would be optimal for this sub-group of patients are unclear. However, the omission of malnutrition and CYP2D6 metabolizer status is no different from current dosing, and the proposed dosing regimen suggested here will not augment these limitations.

Restricting the number of dosing bands resulted in broad weight bands of 17–40 kg and 41–80 kg, and a relatively low total dose of 5.25 mg/kg as well as high mg/kg dose that increased with decreasing age. Unlike previous work on SLDPQ [34, 35, 54], ‘optimal’ therapeutic dosing ranges of daily primaquine for different weight/age bands were not defined because of the paucity of efficacy, safety and pharmacokinetic data in children < 5 years, the differential risk of primaquine-induced haemolysis by G6PD status, and the risk benefit ratio once reliable field-adapted G6PD tests become available. Nevertheless, limited evidence, chiefly in adults, supports a total dose of 5.25 mg/kg as the minimum effective dose [17, 21, 22] and a probable maximum tolerated dose of 1 mg/kg/day, given its risks of blood transfusion in G6PD heterozygous females with South East Asian G6PD variants [33] and high rates of abdominal pain [4, 5, 27, 28].

The proposed dosing could be optimized further by using more dosing bands for both weight-based and age-based regimens, while still using the same tablet strengths, but they might be less user-friendly for patients and health workers. Accordingly, the needs of MCPs should be expressed clearly and channelled through the WHO so a consensus on dosing can be reached to put an end to the multiplicity of regimens and the vagueness of a simple mg/kg target dose.

The study reported herein has several limitations. Firstly, the weight-based regimen presented is based on a model that was constructed using adult pharmacokinetic data, which was adjusted for body weight but not for CYP2D6 status or maturation of physiological processes that might affect the pharmacokinetic properties. Although the anthropometric database was large, 70% of subjects were female and a fifth were children < 5 years, because the predominance of the DHS data; in addition, the total number of heavy individuals was small. Extending DHA data collection to include older children of both sexes would be a helpful step. Our database was from the GMS; therefore, the age-based dosing regimen cannot be generalized to other regions because anthropometric characteristics vary by region [55]. Finally, dosing based on body surface area was not considered but this approach is currently not realistic in most malaria endemic settings where 8-aminoquinoline regimes are used.

## Conclusions

Work presented here proposes a weight-based and an age-based dosing radical cure regimen of primaquine for tropical *P. vivax*, using tablet strengths called for by the WHO and already proposed in regimens of SLDPQ. The age-based dosing showed substantially more variability in expected exposures and should be used only if weight-based dosing cannot be implemented. Young children bear the brunt of the *P. vivax* burden in endemic areas as they have relatively little disease controlling immunity. Relapses are frequent and associated with significant morbidity. These dose recommendations for primaquine radical cure based largely on adult data should be regarded as provisional pending further information on the pharmacokinetic properties of primaquine in younger children. If the trends observed in African children given SLDPQ  are confirmed in young children receiving radical curative regimens for vivax malaria, and there is evidence for correspondingly reduced bioactivation of primaquine, then doses will need to be increased.

## Supplementary Information

**Additional file 1: Table S1.** Primaquine regimens for radical cure of *P. vivax* malaria used in studies and recommended by malaria control programs.

**Additional file 2: Figure S1 & S2.** Weight-for-age growth curves for the Greater Mekong Subregion up to 60 and 15 years of age.

**Additional file 3: Figure S3.** Weight distributions, stratified by sex and disease status in the four age categories.

**Additional file 4: Figure S4.** The mg/kg daily doses predicted for the weight-based and age-based regimens.

**Additional file 5: Table S2.** Breakdown of the mg/kg daily dose for each age in the age-based regimen.

## Data Availability

Selected data generated and analysed during this study are included in this published article and its supplementary information files. Requests for additional data can be made in the first instance to the corresponding author whose institution has a data access committee that considers requests for data.

## Abbreviations

CYP: Cytochrome
DHS: Demographic Health Survey
G6PDd: Glucose-6-phosphate dehydrogenase deficiency
GMS: Greater Mekong Sub Region
IMPROV: Improving the Radical Cure of Vivax Malaria
n: Normal
MCP: Malaria Control Programme
SLDPQ: Single low dose primaquine
WHO: World Health Organization
