# Supplementary material for: Development of weight and age-based dosing of daily primaquine for radical cure of vivax malaria
Source: Malar J. 2021 Sep 9;20:366. doi: 10.1186/s12936-021-03886-w (PMC8427859; doi:10.1186/s12936-021-03886-w)
Supplement: Supplementary file 1 — Additional file 1: Table S1. Primaquine regimens for radical cure of P. vivax malaria used in studies and recommended by malaria control programs. [file 12936_2021_3886_MOESM1_ESM.pdf]

Appendix 1: Primaquine regimens for radical cure of *P. vivax* malaria, used in studies and recommended by malaria control programs

Best Primaquine dose study [1].

For children

Weight (Kg)	mL	Dose in mg	mg/kg dose	total mg/kg dose in 14 d
5	0.8 ml	2.4	0.48	6.72
6	1.0 ml	3	0.5	7
7	1.2 ml	3.6	0.51	7.2
8	1.3 ml	3.9	0.49	6.83
9	1.5 ml	4.5	0.5	7
10	1.7 ml	5.1	0.51	7.14
11	1.8 ml	5.4	0.49	6.87
12	2 ml	6	0.5	7
13	2.2 ml	6.6	0.51	7.11
14	2.3 ml	6.9	0.49	6.9
15	2.5 ml	7.5	0.5	7
16	2.5 ml	7.5	0.47	6.56
17	2.5 ml	7.5	0.44	6.18
18	3 ml	9	0.5	7
19	3 ml	9	0.47	6.63
20	3 ml	9	0.45	6.3

Each tablet contains 15 mg. Dose: (0.5 mg/kg/day) for 14 days. A suspension is made by allowing 1 tablet to dissolve in 5ml clean water (1ml=3mg)

For adults

Weight (Kg)	N tabs	Dose in mg	mg/kg dose	total mg dose in 14 d
21 - 26	0.75	11.25	0.43 - 0.54	6.06 - 7.5
27 - 34	1	15	0.44 - 0.56	6.18 - 7.78
35 - 40	1.25	18.75	0.47 - 0.54	6.56 - 7.5
41 - 48	1.5	22.5	0.47 - 0.55	6.56 - 7.68
49 - 56	1.75	26.25	0.47 - 0.54	6.56 - 7.5
57 - 65	2	30	0.46 - 0.53	6.46 - 7.37
66 - 80	2.5	37.5	0.47 - 0.57	6.56 - 7.95
81 - 100	3	45	0.45 - 0.56	6.3 - 7.78

IMPROV study regimen [2]

Weight/kg	0.5 mg/kg/day regimen	mg/kg /day	Total dose in mg/kg in 14 days	1 mg/kg/day regimen	mg/kg/day	Total dose in mg/kg in 7 days
5 to 22	see suspension dosing chart					
23-34	15	0.44 - 0.65	6.2 - 9.1	30	0.88 - 1.3	6.2 - 9.1
35-45	22.5	0.5 - 0.64	7.0 - 9.0	45	1 - 1.29	7.0 - 9.0
≥ 46	30	≤ 0.67	≤ 9.4	60	≤ 1.33	≤ 9.4

Primaquine suspension dosing chart.

A primaquine tablet is crushed and suspended.

1) For children randomised to receive 14 days primaquine provide 3 mg PQ in 1ml suspension (e.g. one 15mg tablet in 5ml syrup→ 1ml=3mg).

1ml=3mg

Weight	ml/day	Actual dose given (mg)	Actual dose given mg/kg/day	Total mg/kg dose in 14 days
5	0.8	2.4	0.48	6.72
6	1	3	0.5	7
7	1.2	3.6	0.51	7.2
8	1.3	3.9	0.49	6.83
9	1.5	4.5	0.5	7
10	1.7	5.1	0.51	7.14
11	1.8	5.4	0.49	6.87
12	2	6	0.5	7
13	2.2	6.6	0.51	7.11
14	2.3	6.9	0.49	6.9
15	2.5	7.5	0.5	7
16	2.5	7.5	0.47	6.56
17	3	9	0.53	7.4
18	3	9	0.5	7
19	3	9	0.47	6.63
20	3	9	0.45	6.3
21	3.5	10.5	0.5	7
22	3.5	10.5	0.48	6.68

2) For children randomised to receive **7 days primaquine** provide 6mg PQ in 1ml suspension (e.g. two 15mg tablets in 5ml syrup → 1ml=6mg, based on the properties of the primaquine tablets other concentrations may need to be explored).

1ml=6mg

**Weight**	**ml/day**	**Actual dose given (mg)**	**Actual dose given mg/kg/day**	**Total mg/kg dose in 7 days**
5	0.8	4.8	0.96	6.72
6	1	6	1	7
7	1.2	7.2	1.03	7.2
8	1.3	7.8	0.98	6.83
9	1.5	9	1	7
10	1.7	10.2	1.02	7.14
11	1.8	10.8	0.98	6.87
12	2	12	1	7
13	2.2	13.2	1.02	7.11
14	2.3	13.8	0.99	6.9
15	2.5	15	1	7
16	2.5	15	0.94	6.56
17	3	18	1.06	7.41
18	3	18	1	7
19	3	18	0.95	6.63
20	3	18	0.9	6.3
21	3.5	21	1	7
22	3.5	21	0.95	6.68

Regimen used by Wagchuch et al in Bhutan [3].

Body weight	Dose given / day in mg	mg/kg/day	total dose in mg/kg in 14 days
5 - 14	2.5	0.18 - 0.5	2.52- 7
15 - 24	5	0.16 - 0.25	2.24 - 3.5
25 - 34	15	0.22 - 0.3	3.08 - 4.2
35 - ≥ 60	30	≤ 0.25 - 0.43	3.5 - 6.02

Regimen used by Takeuchi et al in Thailand [4].

Patients were dosed by age. We have used our modelled weight for age growth curves to estimate the minimum and maximum weights for each age to estimate the mg/kg dose received.

Age	Weight kg	Dose mg	mg/kg/day	total dose in mg/kg in 14 days
3 - 7	8.3 - 27	5	0.19 - 0.6	2.66 - 8.4
8 - 13	15 - 49	10	0.2 - 0.67	2.8 - 9.38
≥ 14	25 - 86	15	0.17 - 0.6	2.38 – 8.4

Vietnamese National Guidelines

http://www.impe-qn.org.vn/impe-qn/vn/portal/InfoDetail.jsp?area=58&cat=1066&ID=1322

The recommended regimen currently is 0.25 mg/kg/d for 14 days. Dosing is by age. We have used our modelled age growth curves to estimate the minimum and maximum weights for each age to estimate the mg/kg dose received.

Age	Weight kg	Dose in mg	7.5 mg tablets	mg/kg/day	total dose in mg/kg in 14 days
6m - <3	5 - 16.6	1.87	¼ tablet	0.11 - 0.38	1.54 - 5.32
3 - < 5	8.3 - 21.3	3.75	½ tablet	0.18 - 0.45	2.52 - 6.3
5 - < 12	11 - 42	7.5	1 tablet	0.18 - 0.68	2.5 - 9.52-
12 - < 15	22 - 53	11.25	1½ tablets	0.21 - 0.51	2.94 - 7.14
≥ 15	27 - 86	15	2 tablets	0.17 - 0.56	2.38 - 7.84

Between 2007 and 2009, 0.5 mg/kg/d was recommended for 10 days, for a total dose of 5 mg/kg. Dosing was by age. We have used our modelled weight for age growth curves to estimate the minimum and maximum weights for each age to estimate the mg/kg dose received.

Age	Weight kg	Dose	7.5 mg tablets	mg/kg/d	total dose in 10 d
3 - < 5	8.3 - 21.3	7.5	1	0.35 - 0.9	3.5 - 9
5 - < 12	11 to 42	15	2	0.36 - 1.36	3.6 - 13.6
12 - < 15	22 - 53	22.5	3	0.42 - 1.02	4.2 - 10.2
≥ 15	27 - 86	30	4	0.35 - 1.11	3.5 - 11.1

Regimen recommended in Brazil in 2020 over 7 days

http://bvsms.saude.gov.br/bvs/publicacoes/guia_pratico_malaria.pdf

TABELA 2 – Tratamento de malária por *P. vivax* ou *P. ovale* – OPÇÃO 2

IDADE/PESO	DIA 1		DIA 2		DIA 3		DIA 4	DIA 5	DIA 6	DIA 7
<6 meses <5Kg	25/50		25/50		25/50					
6-11 meses 5-9Kg	25/50	5	25/50	5	25/50	5	5	5	5	5
1-3 anos 10-14Kg	CQ	5 5	CQ	5 5	CQ	5 5	5 5	5 5	5 5	5 5
4-8 anos 15-24Kg	CQ CQ	15	CQ	15	CQ	15	15	15	15	15
9-11 anos 25-34Kg	CQ CQ	15	CQ CQ	15	CQ CQ	15	15	15	15	15
12-14 anos 35-49Kg	CQ CQ CQ	15 15	CQ CQ CQ	15 15	CQ CQ CQ	15 15	15 15	15 15	15 15	15 15
>15 anos 50-69Kg	CQ CQ CQ CQ	15 15	CQ CQ CQ	15 15	CQ CQ CQ	15 15	15 15	15 15	15 15	15 15
70-89Kg	CQ CQ CQ CQ	15 15 15	CQ CQ CQ	15 15 15	CQ CQ CQ	15 15 15	15 15 15	15 15 15	15 15 15	15 15 15
90-120Kg	CQ CQ CQ CQ	15 15 15 15	CQ CQ CQ	15 15 15 15	CQ CQ CQ	15 15 15 15	15 15 15 15	15 15 15 15	15 15 15 15	15 15 15 15

CQ Cloroquina 150 mg; 25/50 Artesunato 25 mg + Mefloquina 50 mg; 5 Primaquina 5 mg; 15 Primaquina 15 mg

IMPORTANTE: • Gestantes, puérperas até um mês de lactação e crianças menores de 6 meses não podem usar a primaquina. • Pacientes que pesem mais de 120 kg (não contemplados nessa tabela) devem ter sua dose de primaquina calculada pelo peso. • Caso surja urina escura, icterícia, pele e olhos amarelos, tontura ou falta de ar, buscar urgentemente auxílio médico. • Sempre que possível, supervisionar o tratamento. • Administrar os medicamentos preferencialmente após as refeições.

Weight kg	Dose in mg/d	mg/kg/d	total dose in mg/kg in 7 days
5 - 9	5	0.56 - 1.0	3.89 - 7
10 - 14	10	0.71 – 0.94	5 to 7
15 - 34	15	0.44 - 1.0	3.09 - 7
35 - 49	30	0.61 - 0.86	4.29 - 6
50 - 69	30	0.43 - 0.75	3.04 - 4.2
70 - 89	45	0.51 - 0.64	3.54 - 4.5
90-120	60	0.5 - 0.67	3.5 - 4.67

Previous regimen in Brazil in children [5]

Age in y	Weight kg	Total dose over 7d mg	Mg/kg total dose in 7 days
1 - 3	10 to 14	40	2.86 – 4.0
4 - 8	15 to 24	70	2.92 - 4.67
9 - 11	25 - 34	105	3.09 - 4.2
12 - 14	35 - 49	150	3.06 – 4.2

Regimen used in Colombia

https://www.paho.org/col/index.php?option=com_docman&view=document&layout=default&alias=1220-guia-para-la-atencion-clinica-integral-del-paciente-con-malaria&category_slug=publicaciones-ops-oms-colombia&Itemid=688

Edad/ peso	Número de tabletas por medicamento por día						
	1 er dia		2 do dia		3 er dia		4-14
	CQ	PQ	CQ	PQ	CQ	PQ	PQ
6-11 m 5-9 kg	1/2	*	1/4	*	¼	*	*
1-3 años 10-14 kg	1	1* Tab5 mg	1/2	½* Tab5 mg	½	½* Tab5 mg	½* Tab5mg
4-8 años 15-24 kg	1	1 Tab5 mg	1	1 Tab5 mg	1	1 Tab5 mg	1 Tab5mg
9 -11 años 25-34 kg	2	1/2 Tab1 5mg	2	1/2 Tab1 5mg	2	1/2 Tab1 5mg	1/2 Tab15mg
12-14años 35-49 kg	3	1 Tab1 5mg	2	1 Tab1 5mg	2	1 Tab1 5mg	1/2 Tab15mg
> 15 años >50 kg	4	1 Tab1 5mg	3	1 Tab1 5mg	3	1 Tab1 5mg	1 Tab15mg

*No administrar en menores de 2 años

Regimen used in Honduras

http://www.bvs.hn/Honduras/salud/norma.de.malaria.en.honduras.pdf

Anexo 1: Tratamiento de la Malaria con Medicamentos de Primera Línea

Cuadro 1

Tratamiento de la Malaria No Complicada por Plasmodium *vivax* y Plasmodium *ovale*

Grupo de Edad	N° comprimidos de Cloroquina. 150 mg			N° de comprimidos de Primaquina por 14 días	
	Día 1	Día 2	Día 3	5 mg	15 mg
< 6 meses (<6Kg)	½	¼	¼		
6 – 11 meses (6-10 kg)	½	½	½	½ (2.5mg.)	
1 - 2 años (10 -14 kg)	1	1	0.5	½ (2.5mg.)	
3 a 4 años (15-18 kg)	1	1	1	1 (5 mg.)	
5 a 7 años (19 – 24 kg)	1.5	1.5	1	1 (5 mg.)	
8 – 10 años (25 -36 Kg	2.5	2.5	1	0	½ (7.5 mg.)
11 – 13 años (37-48 Kg)	3	3	2	0	1 (15 mg.)
14 y más años (>49 kg)	4	3	3	0	1 (15 mg.)

Regimen used in Ethiopia

Annex F. **Primaquine phosphate dose: 0.25 mg base per kg daily schedule for 14 days for *P. vivax***

Weight (kg)	Age (years)	Number of tablets per day for 14 days	
		7.5 mg tablet	15 mg tablet
8-14	7months – 3 years	½ tab	-
15 -18	4 – 5 years	½ tab	-
19 – 24	5 – 7 years	¾	-
25 – 35	8 – 10 years	1	½
36 – 50	11 – 13 years	1 ½	1
50+	14+ years	2	1.5

References

1. Chu CS, Phyo AP, Turner C, Win HH, Poe NP, Yotyingaphiram W, Thinraow S, Wilairisak P, Raksapraidee R, Carrara VI *et al*: **Chloroquine Versus Dihydroartemisinin-Piperaquine With Standard High-dose Primaquine Given Either for 7 Days or 14 Days in Plasmodium vivax Malaria**. *Clinical infectious diseases : an official publication of the Infectious Diseases Society of America* 2019, **68**(8):1311-1319.
2. Taylor WRJ, Thriemer K, von Seidlein L, Yuentrakul P, Assawariyathipat T, Assefa A, Auburn S, Chand K, Chau NH, Cheah PY *et al*: **Short-course primaquine for the radical cure of Plasmodium vivax malaria: a multicentre, randomised, placebo-controlled non-inferiority trial**. *Lancet* 2019.
3. Wangchuk S, Drukpa T, Penjor K, Peldon T, Dorjey Y, Dorji K, Chhetri V, Trimarsanto H, To S, Murphy A *et al*: **Where chloroquine still works: the genetic make-up and susceptibility of Plasmodium vivax to chloroquine plus primaquine in Bhutan**. *Malar J* 2016, **15**(1):277.
4. Takeuchi R, Lawpoolsri S, Imwong M, Kobayashi J, Kaewkungwal J, Pukrittayakamee S, Puangsa-art S, Thanyavanich N, Maneeboonyang W, Day NP *et al*: **Directly-observed therapy (DOT) for the radical 14-day primaquine treatment of Plasmodium vivax malaria on the Thai-Myanmar border**. *Malar J* 2010, **9**:308.
5. Vieira M, Matos Lopes TR, Mello A, de Sena LWP, Commons RJ, Vieira JLF: **Doses of primaquine administered to children with Plasmodium vivax according to an age-based dose regimen**. *Pathog Glob Health* 2020, **114**(7):388-392.
