# Supplementary figures and images for: Development of weight and age-based dosing of daily primaquine for radical cure of vivax malaria
Source: Malar J. 2021 Sep 9;20:366. doi: 10.1186/s12936-021-03886-w (PMC8427859; doi:10.1186/s12936-021-03886-w)

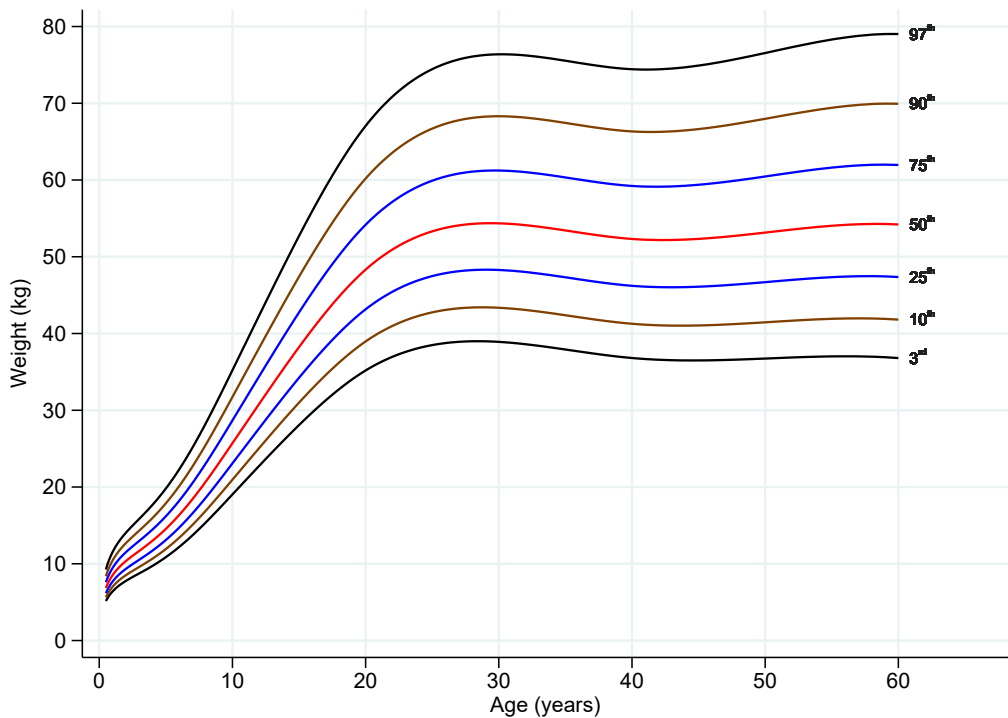

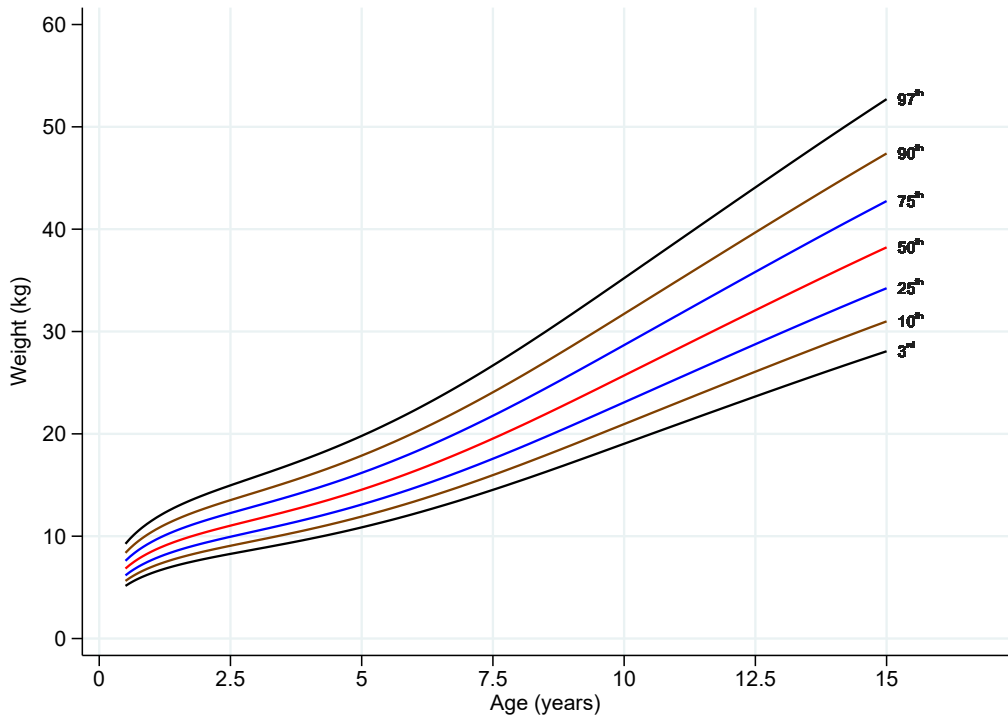

Supplement: Supplementary file 2 — Additional file 2: Figure S1 & S2. Weight-for-age growth curves for the Greater Mekong Subregion up to 60 and 15 years of age. [file 12936_2021_3886_MOESM2_ESM.pdf]

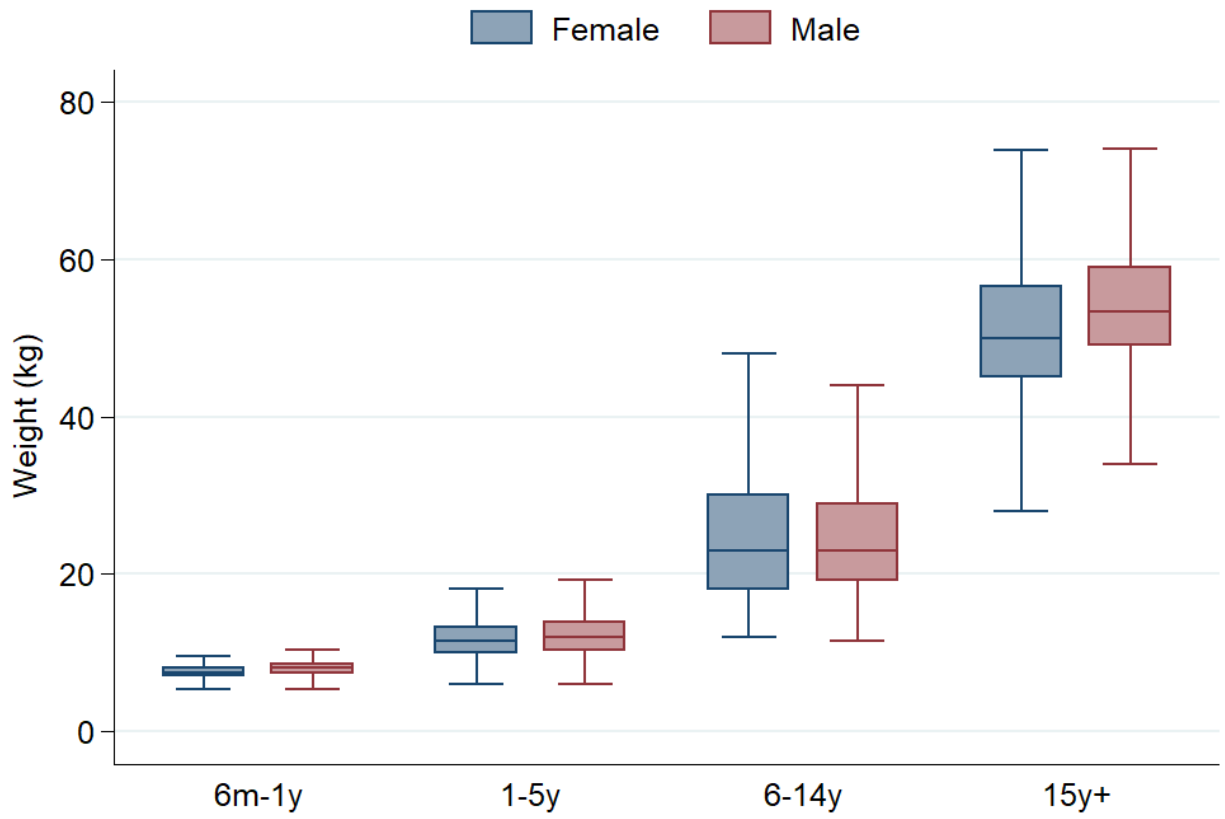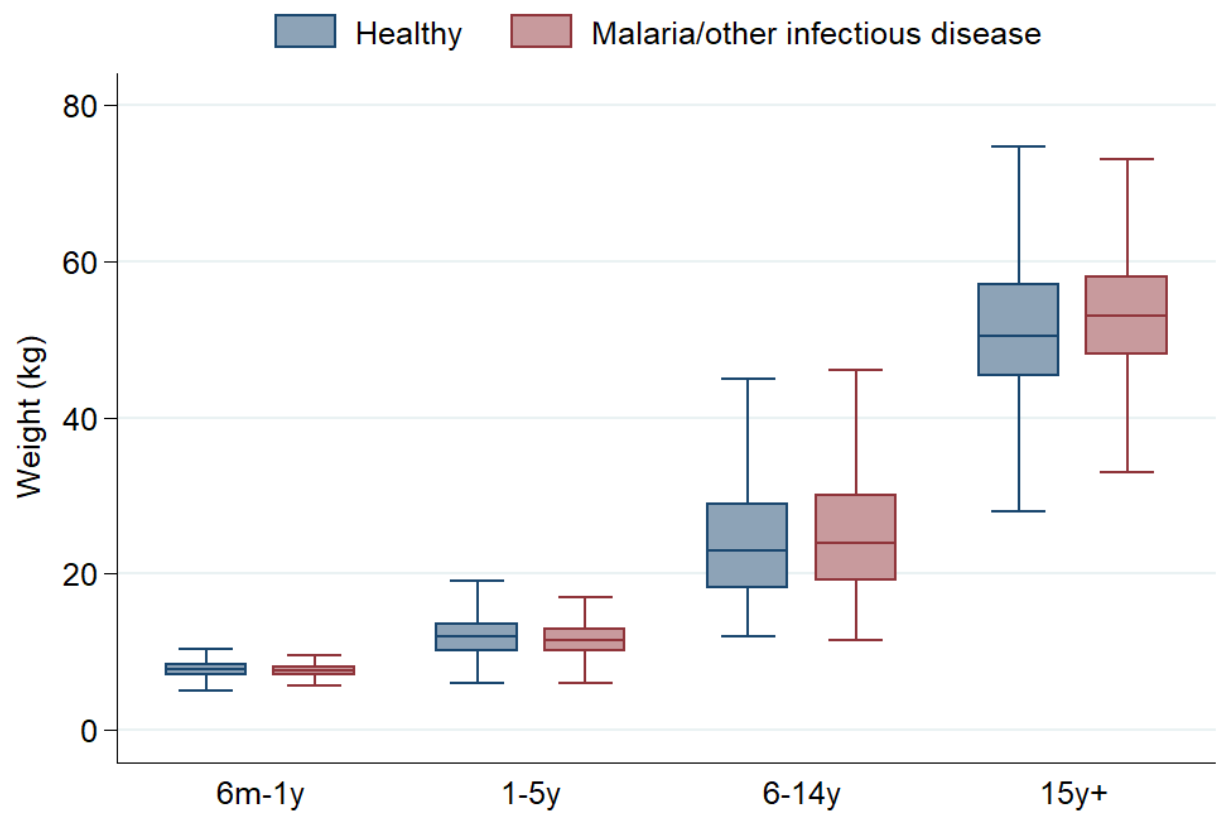

Supplement: Supplementary file 3 — Additional file 3: Figure S3. Weight distributions, stratified by sex and disease status in the four age categories. [file 12936_2021_3886_MOESM3_ESM.pdf]

**Weight-based dosing**

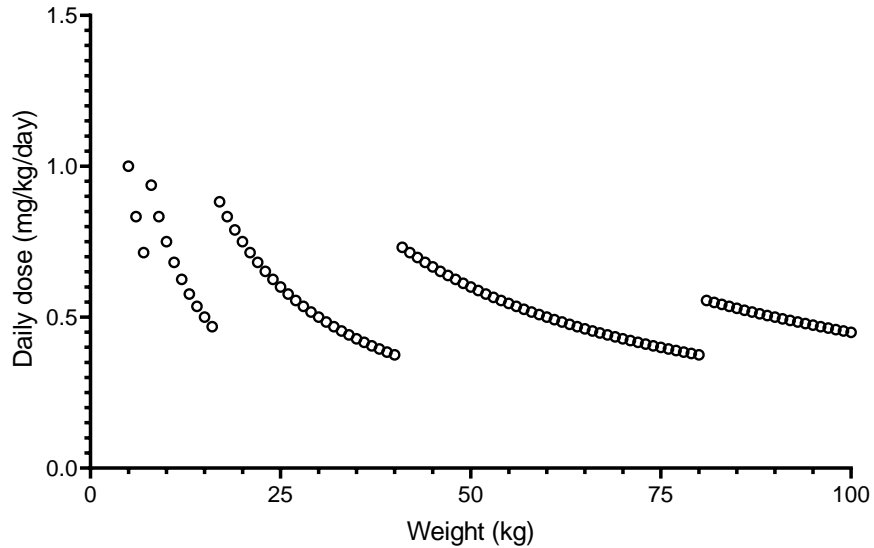

**Age-based dosing**

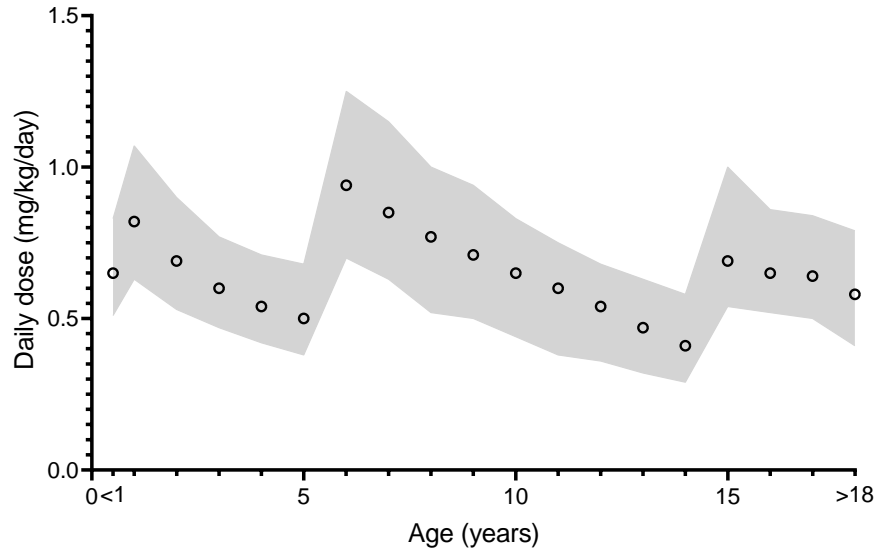

Supplement: Supplementary file 4 — Additional file 4: Figure S4. The mg/kg daily doses predicted for the weight-based and age-based regimens. [file 12936_2021_3886_MOESM4_ESM.pdf]
