# Supplementary material for: Development of weight and age-based dosing of daily primaquine for radical cure of vivax malaria
Source: Malar J. 2021 Sep 9;20:366. doi: 10.1186/s12936-021-03886-w (PMC8427859; doi:10.1186/s12936-021-03886-w)
Supplement: Supplementary file 5 — Additional file 5: Table S2. Breakdown of the mg/kg daily dose for each age in the age-based regimen. [file 12936_2021_3886_MOESM5_ESM.pdf]

Table S2. Detailed breakdown of the mg/kg/day dose of primaquine individuals are predicted to receive as a function of age.

Age band (years)	N	Dose (mg)	Dose (mg/kg/day)						
			Min	P5	P25	P50	P75	P95	Max
<1*	1063	5	0.43	0.52	0.6	0.65	0.71	0.81	1
1	2352	7.5	0.55	0.66	0.75	0.82	0.9	1.04	1.25
2	2426	7.5	0.45	0.55	0.63	0.69	0.75	0.85	1.03
3	2490	7.5	0.4	0.49	0.56	0.6	0.65	0.75	0.91
4	2462	7.5	0.36	0.44	0.5	0.54	0.59	0.68	0.81
5	566	7.5	0.34	0.42	0.47	0.5	0.58	0.65	0.68
6	546	15	0.6	0.75	0.86	0.94	1	1.15	1.3
7	647	15	0.56	0.65	0.76	0.85	0.94	1.07	1.16
8	536	15	0.48	0.58	0.68	0.77	0.83	1	1.03
9	476	15	0.44	0.54	0.64	0.71	0.78	0.88	0.94
10	529	15	0.38	0.5	0.59	0.65	0.71	0.79	0.84
11	489	15	0.36	0.43	0.52	0.6	0.65	0.75	0.77
12	523	15	0.33	0.38	0.47	0.54	0.6	0.66	0.71
13	504	15	0.31	0.33	0.4	0.47	0.54	0.6	0.65
14	472	15	0.28	0.31	0.36	0.41	0.47	0.56	0.6
15†	1209	30	0.52	0.56	0.63	0.69	0.77	0.91	1.11
16	1197	30	0.48	0.54	0.6	0.65	0.71	0.82	1
17	1180	30	0.47	0.52	0.58	0.64	0.7	0.8	1
≥18	33800	30	0.35	0.44	0.52	0.58	0.65	0.75	1.07

* Patients < 1 years of age in the data base included data on patients between 6 and 11 months.

† According to the weight age relationship, individuals aged 15 fell within the 15 mg weight and but their calculated mg/kg daily dose was low: 0.26, 0.28, 0.31, 0.34, 0.38, 0.45 and 0.56.
